# Phylogeny and virulence divergency analyses of *Toxoplasma gondii* isolates from China

**DOI:** 10.1186/1756-3305-7-133

**Published:** 2014-03-28

**Authors:** Min Li, Xu-Wei Mo, Lin Wang, He Chen, Qing-Li Luo, Hui-Qin Wen, Wei Wei, Ai-Mei Zhang, Jian Du, Fang-Li Lu, Zhao-Rong Lun, Ji-Long Shen

**Affiliations:** 1 Anhui Provincial Laboratories of Pathogen Biology and Zoonoses, Department of Microbiology and Parasitology, Anhui Medical University, Hefei, Anhui, China; 2 Blood Center of Anhui Province, Hefei, Anhui, China; 3 Clinical Laboratory, the First Affiliated Hospital of Anhui University of Traditional Chinese Medicine, Hefei, Anhui, China; 4 Clinical Laboratory, the First Affiliated Hospital of Anhui Medical University, Hefei, Anhui, China; 5 Department of Blood Transfusion, the First Affiliated Hospital of Anhui Medical University, Hefei, Anhui, China; 6 Department of Immunology, Anhui Medical University, Hefei, Anhui, China; 7 Central Laboratory of Affiliated Provincial Hospital of Anhui Medical University, Hefei, Anhui, China; 8 Department of Biochemistry and Molecular Biology, Anhui Medical University, Hefei, China; 9 Zhongshan School of Medicine, Sun Yat-Sen University, Guangzhou, Guangdong, China; 10 Center for Parasitic Organisms, State Key Laboratory of Biocontrol, School of Life Sciences and Key Laboratory of Tropical Diseases and Control of the Ministry of Education, Sun Yat-Sen University, Guangzhou, Guangdong, China

**Keywords:** *Toxoplasma gondii*, Microsatellite genotyping, Phylogenetic network, Virulence analysis

## Abstract

**Background:**

*Toxoplasma gondii (T. gondii)* is a very successful parasite that can infect virtually all warm blooded animals with a worldwide distribution. It causes a large range of clinical manifestations in both humans and domesticated animals. In addition, marked biological differences exist among *T. gondii* strains in the pathogenicity and geographical distribution. Molecular epidemiology studies primarily based on restriction fragment length polymorphism (RFLP) method revealed that three main types are predominant in North America and Europe, whereas other diverse genotypes are found in other parts of the world. Microsatellite (MS) as a type of genetic marker has been widely used in many organisms. Limited MS genotyping, however, to fingerprint *T. gondii* isolates has been reported and little is known about the MS data of the strains predominantly prevalent in China.

**Methods:**

Genotyping of twenty-eight Chinese *T. gondii* isolates were performed using 15 MS markers located on 12 different chromosomes. Results were analyzed in terms of population structure by a Bayesian statistical approach. Phylogenetic analysis was obtained from a Neighbor-Net phylogenetic network. The virulence analyses of some representative isolates were determined by inoculation of mice and cell invasion assays. The gene expressions of some virulence-associated factors (VFs) were performed by quantitative real-time PCR (qRT- PCR).

**Results:**

Three haplogroups were clustered among the 28 isolates although minor genetic differences were found within haplogroups. The majority of strains belong to one haplogroup corresponding to the previously described Chinese 1 type (ToxoDB#9). Phylogenetic networks uncovered a limited diversity of *T. gondii* strains and the virulence differs in the strains sharing the same genotype. No remarkable difference, however, was noted in the tested VFs except for dense granule protein3 (GRA3), which was found to have a higher expression in low virulent TgCtwh6 (Wh6) strain than that in high virulent TgCtwh3 (Wh3) strain.

**Conclusion:**

The profile of microsatellite typing data from Chinese *T. gondii* strains revealed a limited genetic diversity and the selected VFs and phylogenetic network analyses displayed less divergence, although the strain virulence differs in the Chinese 1 type of *T. gondii* predominantly prevalent in China.

## Background

*Toxoplasma gondii* is an obligate intracellular protozoan that infects virtually all warm-blooded animals. Nearly one-third of the adult human population has been exposed to the parasite worldwide [1]. Human infections are mainly due to ingestion of undercooked meat containing tissue cysts, or by drinking water containing oocysts. The infection can be asymptomatic, severe or even fatal, and cause problems in common health. In addition, *T. gondii* infection can also cause severe damage to livestock and thus lead to a huge economical loss.

The genetic diversity of *T. gondii* in various geographical regions has been widely investigated in the world [2-10]. The first typing study, which described a highly clonal population structure with three major lineages, Type I, II, and III, were performed in North America and Europe. However, in South America, the genetic polymorphisms were more complex and comprised of a large number of distinct genotypes. Recent studies showed the fourth clonal lineage was widely spread among the wildlife in North America [7,11]. Additionally, the main clonal lineage in East Asia, especially in China, has been designated as Chinese 1 genotype (ToxoDB#9) [12,13]. It has been demonstrated that the genetic diversity of *T. gondii* was associated with the distinct symptoms in host [14]. In terms of mouse virulence, Type I isolates are considered as the most virulent, and can lead to death of mice less than 10 days after inoculation; in contrast, strains of Type II and Type III are avirulent and usually cause chronic infection and shaped tissue-cysts. Cystogenic *T. gondii* strains could be divided into different virulent strains, however, all non-cystogenic isolates presented high virulence in mice [15].

Most of the genotyping data of *T. gondii* were based on the conventional method of restriction fragment length polymorphism (RFLP). This approach is rapid and easy to use, but technical problems have been reported, e.g., the incomplete amplification of SAG2 marker [16] and insufficient digestion of amplicon by restriction enzymes [17,18]. Moreover, multilocus studies of RFLP required several PCR assays and enzyme digestions that are time-consuming and procedure complexity. Microsatellites, as a type of genetic marker, are generated from short tandem repeats and known to be highly polymorphic. Although polymorphic through an evolutionary process, these markers are generally stably inherited between closely related individuals. These properties have led to their extensive use in studies of diversity of population structure, and in the determination of lineage and clonality [19-22].

Our previous RFLP analysis revealed a predominant Chinese 1 *T. gondii* lineage prevalent in China [12]. When mice were inoculated with 1000 tachyzoites, some Chinese 1 isolates caused a different severity of manifestations, and certain isolates could generate abundant cysts in the brain of infected mice. Thus far, the underlying reasons for this discrepancy in virulence have not been fully described.

To get a better understanding of the genetic background of *T. gondii* collected from a variety of regions in China, we performed the microsatellite genotyping to identify the precise haplotypes among Chinese *T. gondii* populations. Additionally, the mouse virulence of isolates *in vivo* was observed in a dose-dependent way and cell invasion ability *in vitro* was also observed to deeply elucidate the phenotypical characteristics of the virulence difference of the strains sharing the common genotype. Furthermore, an expression profile of VFs was also identified here.

## Methods

### Animal ethics

All procedures carried out on mice were in agreement with ethical permission obtained from the Institutional Review Board (IRB) of the Institute of Biomedicine, Anhui Medical University. The IRB approved both animal experimental operation protocols and handling procedures carried out on the stray cats, in accordance with the regulations from the Care and Use of Laboratory Animals of the National Institutes of Health, China.

### *T. gondii* strain preparation and isolation from bioassay in mice

All stray cats were trapped from several locations of Hubei, Jiangsu, Guangdong, Shanxi, Guizhou and Anhui Provinces of China and anesthetized before being sacrificed and brain, tongue and heart were removed from each cat for *T. gondii* examination and isolation. The retail pork was collected on the markets as previously described [12,13,18,23]. The isolates were harvested from the peritoneal fluids, or the brain tissues of mice, and maintained in the laboratory by mouse passage. All tachyzoites were diluted with PBS and then inoculated with 4–6 week old Kunming mice (Anhui Laboratory Animal Center). The parasites were collected from the peritoneal fluid of infected mice.

### Genetic analysis of *T. gondii* isolates

#### Microsatellite genotyping

*T. gondii* DNA extraction was carried out using the commercial QIAamp® DNA Mini kit (QIAGEN, Germany) according to the instructions. In total, 28 Chinese isolates and 6 reference strains were studied using a multiplex PCR assay with 15 MS markers according to Ajzenberg [24]. These markers were classified into two sorts, eight were typing markers (*TUB2, W35, TgM-A*, *B18, B17, M33, IV. 1* and *XI. 1*), and seven were fingerprinting markers (*M48, M102, N60, N82, AA, N61 and N83*). The forward primers were 5′-end labelled with one kind of fluorescein of 6-FAM, HEX or TAMRA. Multiplex PCR was established using the commercial QIAgen multiplex PCR kit (QIAGEN, Germany) following the manual description. PCR products were sent to Sangon Biotech Company (Shanghai, China) to get the fragment length of the alleles. The *W35* locus was sequenced due to the different tandem repeats with identical lengths of fragments in genotypes.

### Structure analysis

To better understand the population information, we used a Bayesian statistical approach, STRUCTURE version 2.3.4 [25], to cluster Chinese strains and worldwide isolates by MS markers respectively. The worldwide MS raw data were derived from other reports [20,24]. Three simulation runs were calculated for K = 1 to K = 10 using a length of 10,000 burnin period of Markov chain Monte Carlo repetitions. An estimation of the optimal number of clusters, K, was calculated according to the method described by Evanno [26].

### Phylogenetic analysis

Phylogeny networks were constructed using three different levels of resolution markers and the primers were listed in Additional file 1, according to previously published research [27]. Three conserved markers, *AP1*, *AP2* and *AP3*, comprising 1756 bp, are from the apicoplast DNA. Eight normal resolution markers, comprising 2974 bp are from genomic DNA. *W35* marker was designated as the highest resolution marker. Sequences used in the present study have been deposited into GenBank (Accession Nos. KJ159646- KJ159889). Concatenated sequences were analyzed using the Neighbor-Net method implemented in Splits Tree version 4.13.1 application [28].

### Virulence analysis of *T. gondii* isolates from China

#### Dose effect and mouse virulence tests

To precisely identify mouse virulence of representative Chinese isolates, acute virulence was determined by monitoring cumulative mortality after intraperitoneal injection. Six strains (TgCtxz1, 3, 5; TgCtwh3, 6; and TgCtgy1) and two reference strains (RH, PRU) were tested. Different doses of tachyzoites ranging from 10^4^ to 10^0^ of each strain were intraperitoneally inoculated into groups of outbred female Kunming mice (SPF), and observed for 30 days. Some strains were repeated two or more times, the data were representative of one experiment. After the end of the censored point, surviving mice with 10^0^ parasites were bled from the orbital sinus; the sera were detected by Western blotting for antibodies against *T. gondii*. The virulence was determined by cumulative mortality defined as the number of deaths/ the number of mice infected (with tachyzoites or cysts in brain tissues, and/or positive antibodies). The statistical comparison of survival curves,median survival time and hazard ratio were estimated by Log-rank (Mantel-Cox) test performed using GraphPad Prism 5, San Diego, California, USA.

### Cell invasion assays

To further identify the different virulence between the same genotype isolates, we compared the growth rate of *T. gondii* strains *in vitro*. Tachyzoites were maintained *in vitro* by serial passages on host monolayers of human foreskin fibroblasts (HFFs) at 37°C, 5% CO_2_. HFFs were cultured in Dulbecco modified Eagle medium (DMEM, GIBCO) supplemented with 10% newborn calf serum (GIBCO), 2 mM glutamine, 100 U/ml penicillin and 0.1 mg/ml streptomycin. A host cell-parasite ratio of 1:3 was used to observe the cell infection and the intracellular growth of tachyzoites. Each co-cultured experiment was in duplicate. After 2 h, 8 h, 16 h, 24 h and 48 h post infection (p.i.), the co-cultured cells were stained with Wright-Giemsa. At least 20 visual fields (1000 ×) were randomly picked up. Total number of cells in each field, both infected and un-infected, and all tachyzoites in infected cells were counted simultaneously. The cell infection rate % = the number of infected cells/the total number of cells × 100%. The mean of tachyzoites per infection cell = total number of tachyzoites in infected cells/total number of infected cells.

### RNA purification and qRT-PCR for virulence-associated factors of Chinese 1 isolates

To further investigate the cause of the divergent phenotypes among the same genotype strains, we tested the gene expression levels of putative VFs for Wh3 and Wh6. Viable tachyzoites or cysts were freshly collected and total RNAs were purified by TRIzol reagent (Invitrogen, USA). Reverse transcription was performed using RevertAidTM First Strand cDNA Synthesis Kit (Fermentas, Germany). The cDNA products were subject to qRT-PCR with the specific primers for ROP2, ROP4, ROP5, ROP16, ROP18, GRA2, GRA3, GRA5, GRA7, GRA15, MIC6, and *TgGAPDH* as a control using SYBR® Premix Ex TaqTM II (TaKaRa, Japan). Primer sequences were given in Additional file 2. The amount of each target gene, normalized to the endogenous housekeeping gene (*TgGAPDH*), was given according to previously described methods [29,30]. Statistical analysis was performed using One-way ANOVA test and Independent Samples T test.

## Results

### Allelic polymorphism and genetic diversity

Through the fifteen MS markers, 79 different multilocus polymorphisms were identified. The number of alleles per marker varied from 2 to 12, and an average of 5.3 alleles per locus was carried out. Loci *M33*, *B18,* and *IV. 1* showed two alleles. Markers *TUB2*, *B17* displayed three length polymorphisms. *W35* marker had 2 fragment lengths but 4 different tandem repeats: 248 bp of (TC)_10_ (TG)_2_ for Type I allele; 242 bp fragment, (TC)_7_ (TG)_2_ for allele of Type II and ToxoDB #205, (TC)_6_ (TG)_3_ for allele of Type III, (TC)_4_CC(TC)_2_(TG)_2_ for ToxoDB#9 allele (Chinese 1 isolates). The other markers carried more than 4 alleles, in particular 12 alleles for *AA* and 10 alleles for *N82*, indicative of a higher allelic polymorphism.

Overall, a total of 21 different genotypes based on 15 MS loci was found in the population of 28 Chinese animal strains (Additional file 3). Four of them contained two or more isolates, while 17 genotypes comprised only one isolate. In brief, typing markers could distinguish the major clonal lineages from atypical strains although some minor differences existed, however, finger printing markers provided enhanced genetic resolution in recognizing closely related isolates within one clonal lineage or haplogroup.

### Genetic analysis of *T. gondii* isolates

#### Structure clustering analysis

**Figure 1:**
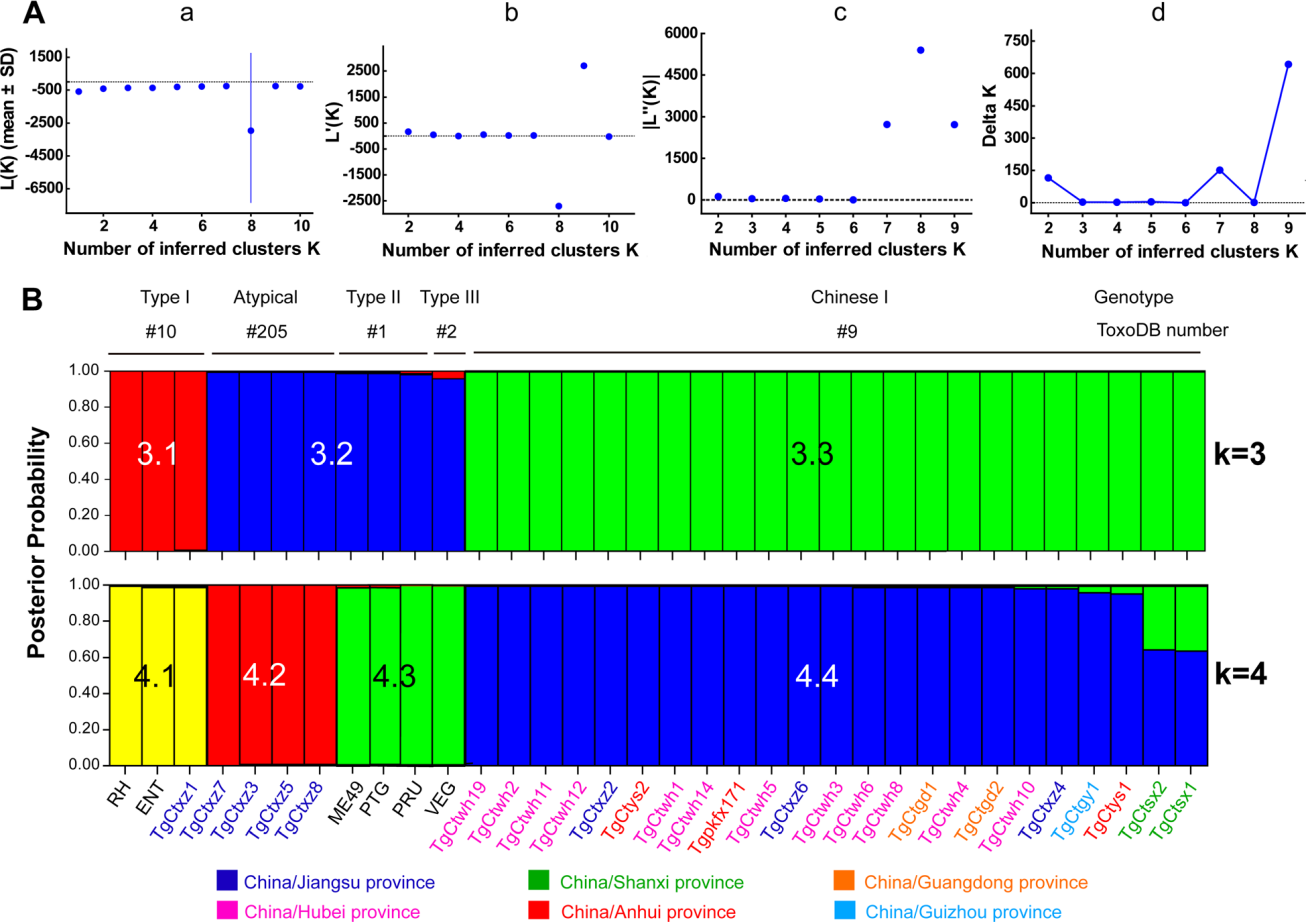
**Clusters by STRUCTURE implemented with 15 microsatellite markers in Chinese *T. gondii *isolates. (A)** Description of the four steps in detection of the true number of clusters K. Aa: *L (K)*, plot of the mean likelihood, mean (± SD) of *Ln P (D)* over 3 runs for successive *K* values of 1 to 10; Ab: *L’(K)*, plot of the mean difference between successive likelihood values of *K*, *L’(K) = L (K) –L (K-1)*; Ac: | *L”(K)* |, plot of the absolute values of the average differences between successive values of *L’(K)*, | *L”(K)* | = | *L’(K + 1)- L’(K)* |; Ad: Delta *K* (*ΔK*) = *m* | *L”(K)* | / *s* [*L (K*)], where *m* = means of the absolute values of *L”(K)*, divided by the standard deviation of *L (K)*. For Chinese *T. gondii* isolates, the optimal number of clusters was 3 according to the calculation of *ΔK*. **(B)** The population structure of Chinese isolates based on group sizes, K = 3 and K = 4. Colors represent a contribution from each presumed ancestral population (red, blue and green for Cluster C3.1, C3.2 and C3.3). Each strain was characterized by ToxoDB number, RFLP genotype and geographic location.

To better understand the genetic relationships among Chinese isolates, we used the STRUCTURE software, a Bayesian clustering method to group them (Figure 1). The nearly true number of K (from *ΔK)* derived from the distribution of *Ln P (D)* indicated that K = 3 is the most likely value of clusters (Figure 1A), indicating that Chinese isolates could be clustered into 3 haplogroups, designated as C3.1, C3.2 and C3.3. In addition, we showed the result of K = 4 with the purpose of comparing the consequence.

Based on the K = 3 clustering results, the group C3.1 was quasi-exclusively constituted by TgCtxz1 isolate and typical Type I strains. Isolates grouped in haplogroup C3.2 included atypical ToxoDB#205 and typical Type II/III strains, although Type III VEG strain shared something in common with C3.1. Haplotype C3.3 was constituted from the rest of Chinese 1 isolates. From K = 3 to K = 4, the main change in classification was due to the separation of cluster C3.2 into two sub-clusters C4.2 and C4.3, which contained ToxoDB#205 isolates and typical II/III strains, respectively.

**Figure 2:**
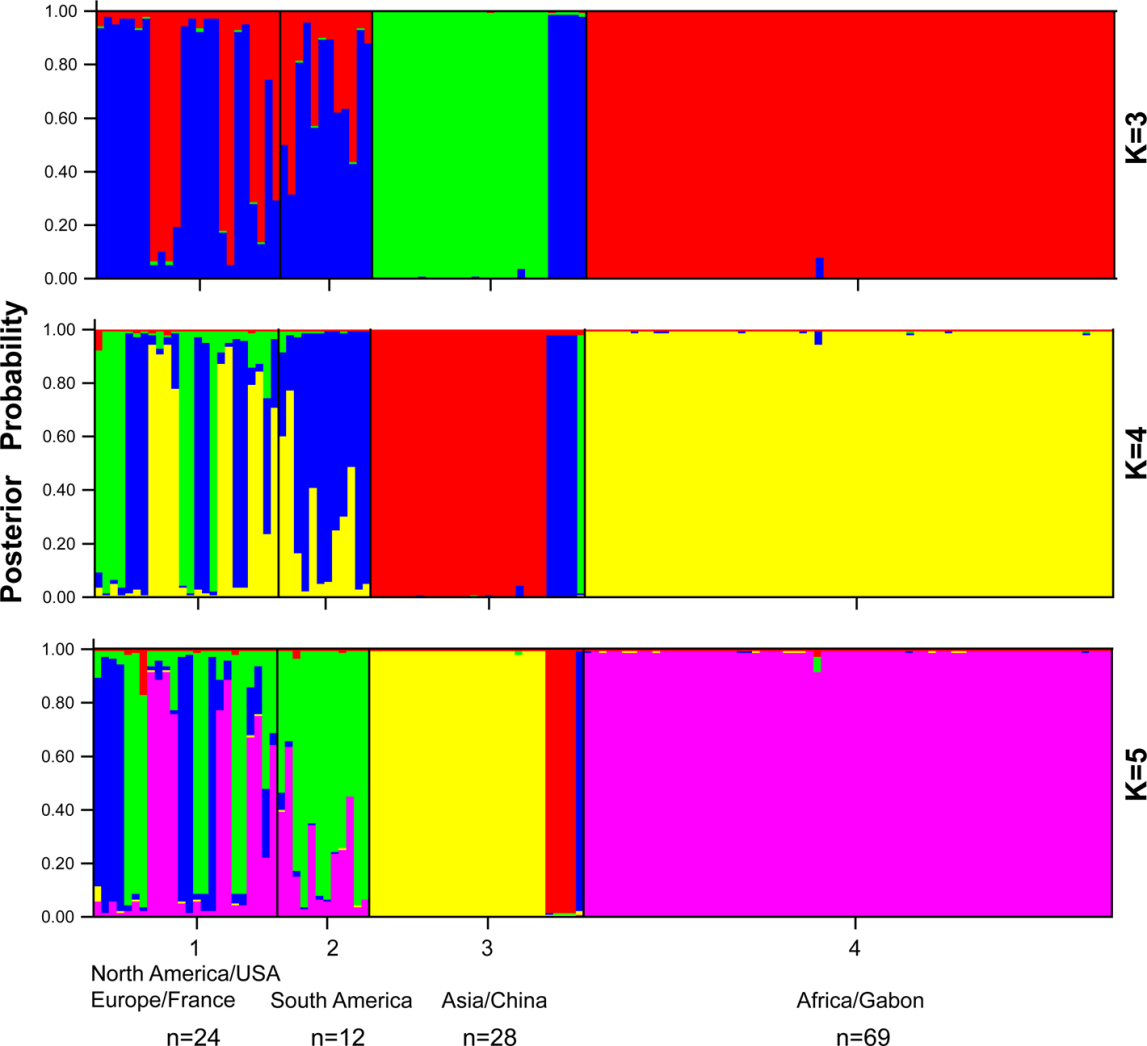
**Population structure in worldwide *T. gondii *isolates.** The optimal K value is 4. Colors represent contributions from different ancestral haplotypes. The results of K = 3 and 5 were also depicted here. The special parameter settings are as follows: Missing data value = −9, POPID setting 1 = North America/ USA and Europe/ France, 2 = South America, 3 = East of Asia/ China, 4 = Africa/ Gabon.

**Figure 3:**
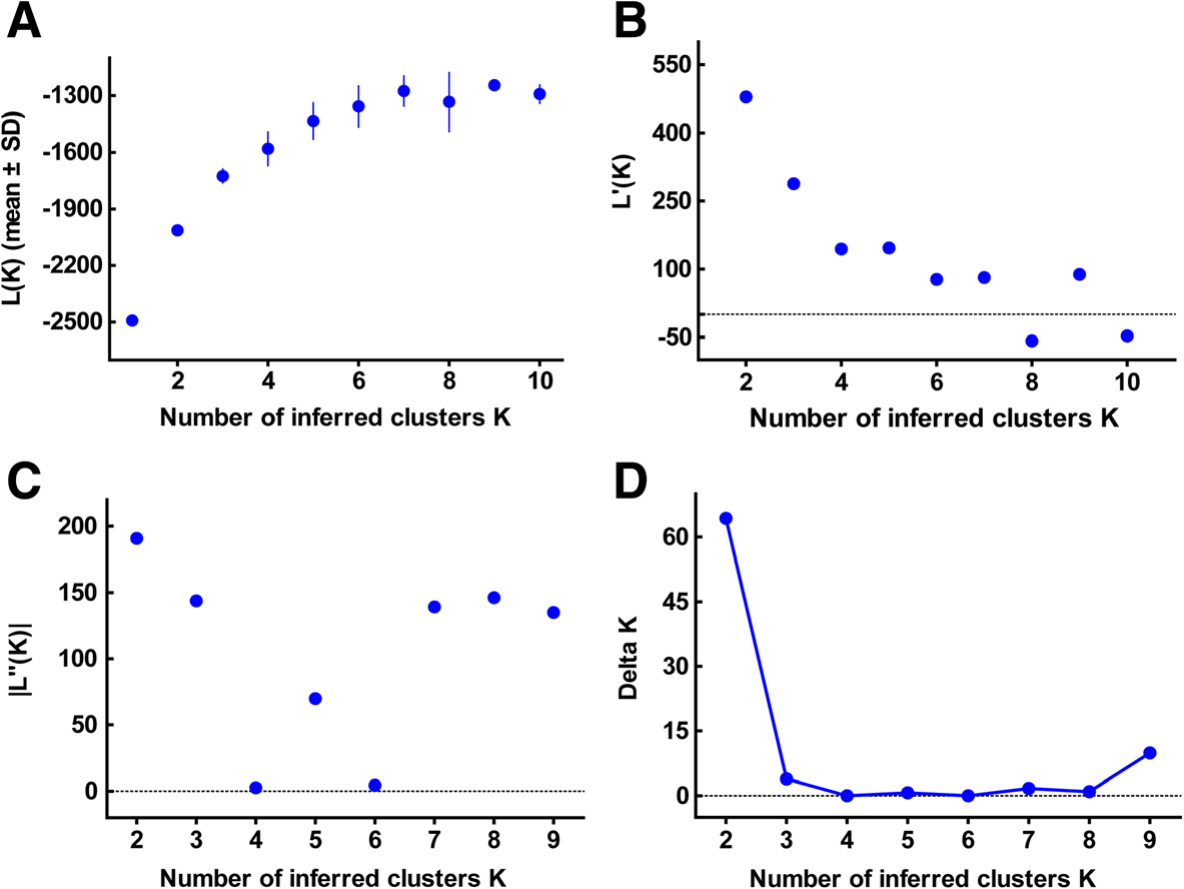
**Determination of the optimal K value among worldwide *T. gondii *isolates.** The calculating formula of *L (K)***(A)**, *L’(K)***(B)**, | *L”(K)* | **(C)** and *ΔK***(D)** were identical to Figure 1A. From the diagram of delta *K*, the most true value of clusters is 4.

In addition, we clustered the Chinese and other continental *T. gondii* isolates [20,24]. As is shown in Figure 2, a total of 133 unique *T. gondii* strains were grouped by geographic origin. From the plot of *ΔK* diagram, we roughly estimated that the true K value was 4 (Figure 3). The results of K = 3 and K = 5 were also displayed here. Based on this combined analysis, Chinese isolates displayed exclusively unique genetic features when compared to other isolates (shown in different colors). Regardless of the clustering numbers, the dominant Chinese 1 strains always displayed a limited diversity, indicating the robustness of clustering ability of the method.

### Phylogenetic analysis

**Figure 4:**
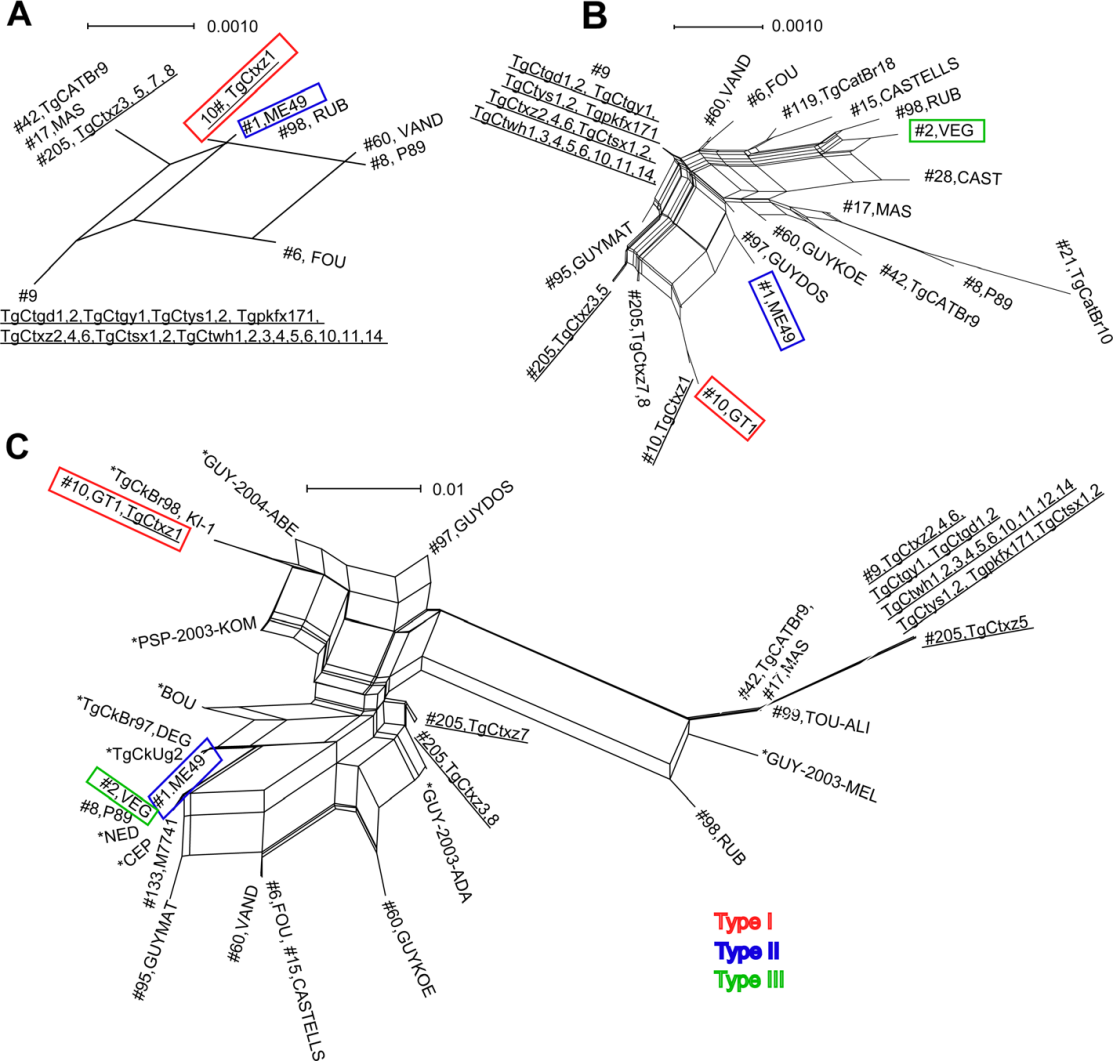
**Phylogeny analyses of *T. gondii *isolates using Splits Tree software. (A)** The result of network completed with relatively conserved apicoplast genome DNA markers. **(B)** The network calculated by the normal mutation frequency of genomic multilocus. **(C)** The Neighbor-Net analysis conducted with the rapidly evolving marker *W35*. Phylogenetic analyses revealed that *W35* marker has highly divergent properties and Chinese isolates possess highly clonal characteristic. *, no accession number from ToxoDB. Scale = substitutions per site.

To have an insight into the evolution and phylogenesis of *T. gondii*, the markers with different mutation frequencies were sequenced and aligned in parallel with those of published strains from GenBank and ToxoDB database (http://toxodb.org/toxo/) (Figure 4). Phylogenetic trees were separately constructed. The apicoplastic sequence networks revealed smaller distance from the archetypal genotype strains. The extent and distribution of variation in *W35* allele were distinct from the other models. Phylogenetic tree constructed by *W35* marker displayed highly divergent branches. In all evolutionary diagrams, Chinese isolates uniformly revealed high clonal characteristics.

### The virulence of Chinese *T. gondii* isolates

#### Mouse virulence tests

**Figure 5:**
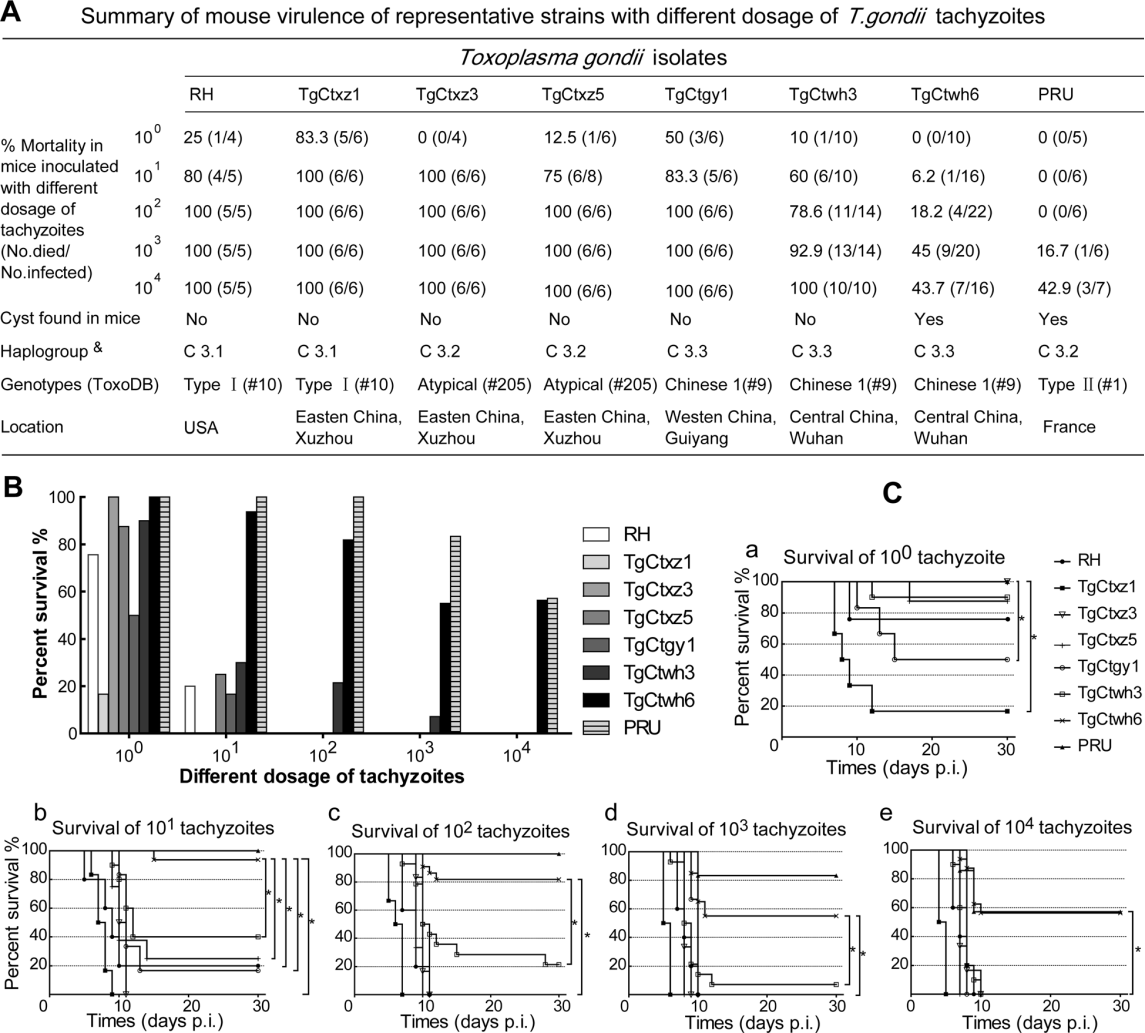
**Characterization of mouse virulence in representative isolates from China. (A)** Summary of mouse virulence with different dosage of tachyzoites. **(B)** Proportion of surviving mice infected with different number of tachyzoites. **(C)** Elaboration of the percentage of survival mice inoculated with equal doses of different isolates in details. &, the corresponding haplogroup (Figure 1, k = 3). *, statistical significance of survival curves between Wh6 and other isolates (P < 0.01).

To identify the virulence of isolates, a series of parasite burdens for each representative strain was given to the mice. Mice that survived from the 10^0^ parasites challenge after the censored point had a positive serologic reaction, indicating the successful inoculation. The mortality and survival of mice post infection are shown in Figure 5. At the inoculum of 10^3^ tachyzoites, most of infected mice died within 30 days except for those infected with Wh6(Chinese 1)and PRU (Type II) strains. Wh6 strain showed low virulence similar to PRU strain and the rest of five Chinese isolates, however, displayed the high virulence consistent with the virulent property of Type I RH and TgCtxz1 strains with haplogroup C3.1 (Figure 5A). The average of survival days post inoculation with 10^4^, 10^3^, 10^2^ and 10^1^ parasite loads was 4.5 to 8 d, 5.5 to 10 d, 6.5 to 10.5 d and 7.5 to 12 d, respectively (Table 1). Comparison of dose-dependent hazard ratio of survival time showed a statistically significant difference between Wh6 and other strains (p < 0.01) (Figure 5B, C and Table 2). The infectious risk of isolates goes in parallel with the dose-adjusted hazard ratio.

**Table 1:** Median survival days of mice inoculated with different doses of parasites

		**Median survival days**							
		**RH**	**TgCtxz1**	**TgCtxz3**	**TgCtxz5**	**TgCtgy1**	**TgCtwh3**	**TgCtwh6**	**PRU**
Dosage	10^0^	Undefined	8.5	Undefined	Undefined	22.5	Undefined	Undefined	Undefined
	10^1^	9	7.5	10.5	10	11	12	Undefined	Undefined
	10^2^	9	6.5	10	9	10.5	10.5	Undefined	Undefined
	10^3^	8	5.5	8	8	10	8.5	Undefined	Undefined
	10^4^	7	4.5	7	7	8	8	Undefined	Undefined

**Table 2:** **P value and hazard ratio (95**% **CI of ratio) for mice survival days estimated from the Log-rank (Mantel-Cox) test**

**Factor**		Hazard Ratio *													
		**RH**		**TgCtxz1**		**TgCtxz3**		**TgCtxz5**		**TgCtgy1**		**TgCtwh3**		**PRU**	
**Dose effect**		**HR(95% CI)**	**P**	**HR(95% CI)**	**P**	**HR(95% CI)**	**P**	**HR(95% CI)**	**P**	**HR(95% CI)**	**P**	**HR(95% CI)**	**P**	**HR(95% CI)**	**P**
**TgCtwh6**	10^0^	66.7(0.67 to 6646)	0.0736	177.5(19.01 to 1658)	< 0.0001	0	1.0	20.1(0.31 to 1284)	0.1573	69.5(4.82 to 1001)	0.0018	13.5(0.24 to 756.5)	0.2059	0	1.0
	10^1^	171.7(14.49 to 2035)	< 0.0001	311.1(37.1 to 2609)	< 0.0001	194.9(24.49 to 1551)	< 0.0001	34.9(5.81 to 209.6)	0.0001	95.8(11.33 to 811.1)	< 0.0001	14.7(2.86 to 75.69)	0.0013	0.3(0.003 to 20.61)	0.5403
	10^2^	597.6(53.35 to 6694)	< 0.0001	889.1(91.75 to 8614)	< 0.0001	104.1(14.46 to 749.6)	< 0.0001	261.0(31.7 to 2149)	< 0.0001	53.2(8.27 to 342.1)	< 0.0001	9.7(3.03 to 30.81)	0.0001	0.3(0.03 to 2.87)	0.2769
	10^3^	69.0 (8.79 to 542.3)	< 0.0001	469.2(52.39 to 4202)	< 0.0001	123.7(16.73 to 915.3)	< 0.0001	225.1(26.93 to 1882)	< 0.0001	8.6(1.77 to 41.38)	0.0076	8.8(3.0 to 25.68)	< 0.0001	0.4(0.09 to 1.8)	0.2278
	10^4^	36.4(5.19 to 255.1)	0.0003	194.9(24.49 to 1551)	< 0.0001	19.0(3.5 to 103.3)	0.0006	61.9(8.64 to 442.8)	< 0.0001	22.4(3.74 to 134.4)	0.0007	11.8(3.33 to 41.61)	0.0001	1.0(0.23 to 4.47)	0.988

*Hazard ratio was adjusted on the number of inoculated parasites.

### Cell invasion assays

**Figure 6:**
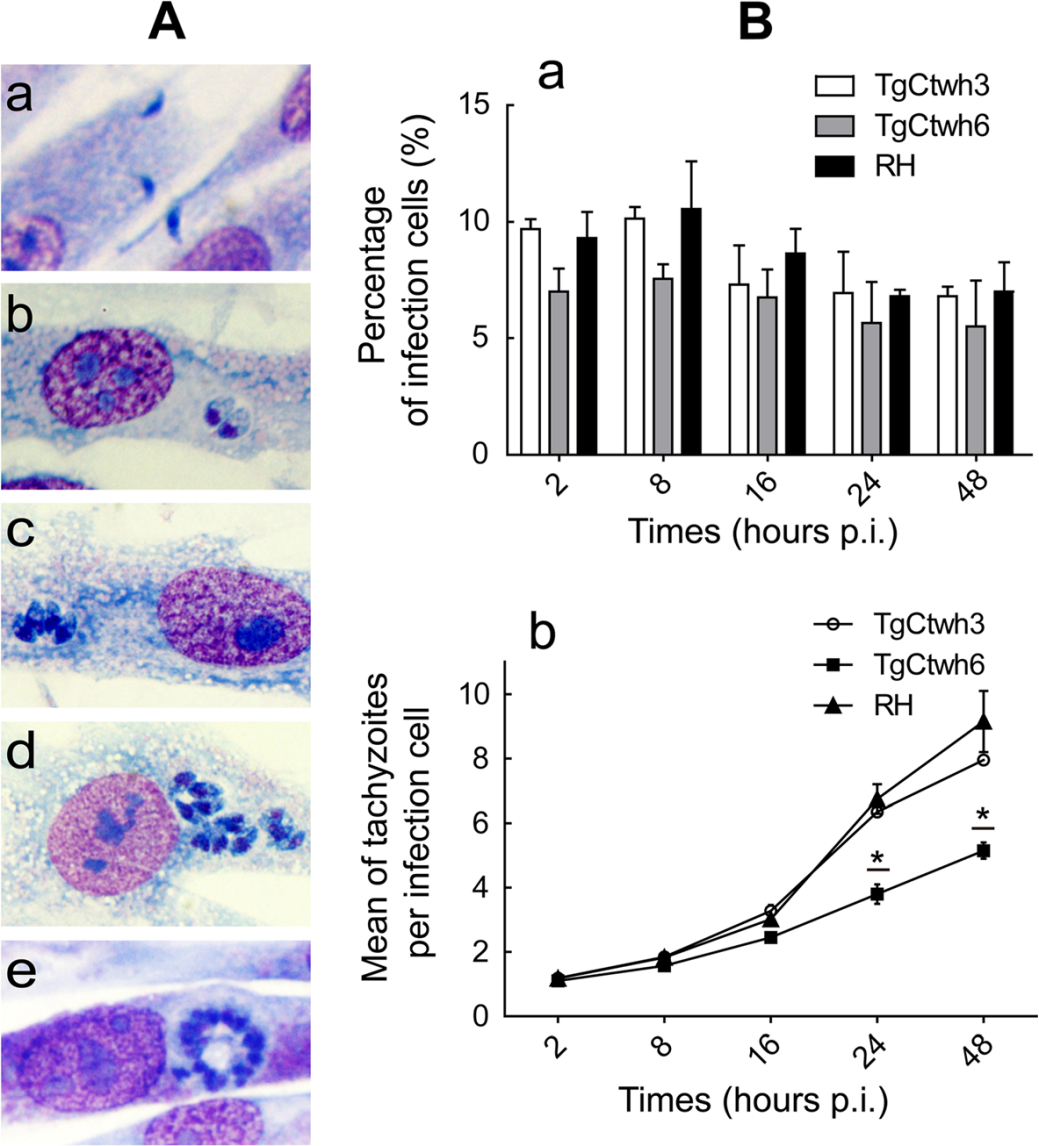
**Cell invasion of haplogroup C3.3** ***T. gondii *isolates. (A)** Propagation of tachyzoites in HFFs with Wright-Giemsa staining: a, b, c, d, and e represent the growth of tachyzoites in host cells after 2 h, 8 h, 16 h, 24 h and 48 h p.i., respectively. **(B)** The growth rate of tachyzoites in infected cells. Ba: the cell infection rate at different post infection time points. There was no significant difference in each time point (P > 0.05); Bb: the mean of *T. gondii* tachyzoites in each infected cell. From the diagram, Wh6 isolate showed a lower growth rate in host cells. *P < 0.01.

Cell invasion and growth rate of Wh3 and Wh6 strains, both belonging to the common haplotype C3.3, were observed compared with RH strain of haplotype C3.1, to clarify their virulent difference. Figure 6A presents parasite duplicates at different time points (2 h, 8 h, 16 h, 24 h, and 48 h p.i.). The remarkable binary fission could be noted from 8 h p.i. and then underwent rapid multiplication. At five time points from 2 h to 48 h p.i., the percentages of infected cells were found to be 9.7%, 10.2%, 7.3%, 7%,6.8% for Wh3, 7.0%, 7.6%, 6.8%, 5.7%, 5.5% for Wh6, and 9.3%, 10.6%, 8.7%, 6.8%, 7.0% for RH, respectively (Figure 6Ba). The result showed that low virulent Wh6 had the invasive capacity to host cells similar to Wh3 and RH (P > 0.05). The average number of parasites in each cell had not been gradually divergent until 24 h p.i., reaching 3.8 parasites in Wh6, 6.4 parasites in Wh3, and 6.7 parasites per cell in RH strain, respectively (Figure 6Bb). Wh6 strain displayed a lower rate of replication than the other two strains (P < 0.01).

### Expression of virulence-associated factors of Chinese *T. gondii* isolates

**Figure 7:**
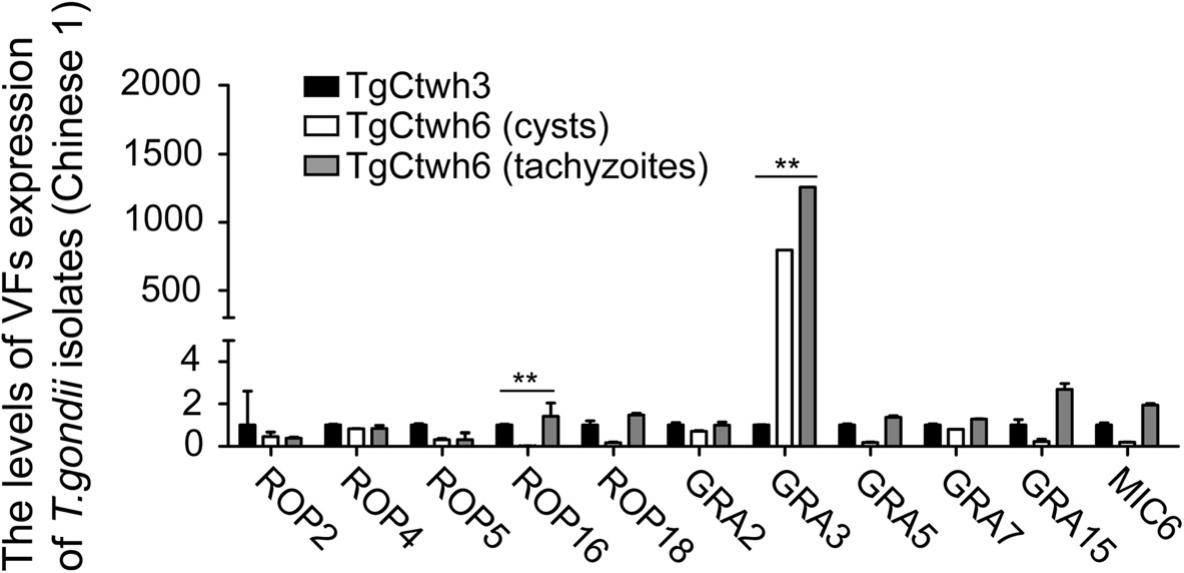
**Gene expression profiles among the isolates with the same haplotype.** It shows a significant increase of GRA3 expression in less virulent Wh6 and ROP16 in virulent Wh3. The qRT-PCR was performed in triplicate and values were reported as means with error bars indicating standard deviation. **P < 0.001.

To fully understand the nature of different virulence, we detected the gene expression of putative VFs of Wh3 and Wh6 isolates by qRT-PCR (Figure 7). A significant increase of ROP16 expression was found in virulent Wh3 isolate (P < 0.001), whereas overexpression of GRA3 was only observed in low virulent Wh6 (P < 0.001). No discrepancy of the rest of VFs were noted between the two strains.

## Discussion

*Toxoplasma gondii* is the only recognized species in the genus *Toxoplasma* and is considered to be one of the most successful organisms of eukaryotic cells in the light of the number of wide host species and percentage of animal infections. It has a prevalence in China of 7.9% of seropositive rate in human populations [31] and 18.0% contamination in retail pork [23]. This imposes a real public risk on human health.

Compared to North America and Europe, the genetic information and population structure of *T. gondii* in China has not been fully explored. Most of the genotyping data were collected based on the RFLP approach [6,8,12,13,32,33]. Here we chose microsatellite markers based on their high mutation rate and being appropriate for study of the population genetic structure [20,34]. Different lengths of MS markers can be easily identified in one PCR assay due to the nature of haploidy of tachyzoites or bradyzoites stage. The data showed that eight typing markers had highly uniform typing patterns not only in archetypical strains but also in most of the atypical Chinese isolates (Additional file 3). The fingerprinting loci provided enhanced genetic resolution in identifying closely related isolates. Unfortunately, the MS-based approach could not draw marked distinction between Wh3 and Wh6, both were depicted as sharing the Chinese 1 genotype and having altered virulence phenotypes in mice [12]. Our results suggested that further transcriptomics and epigenetic technology might be needed to illuminate the genetic background of the virulence difference between Wh3 and Wh6.

According to the MS typing data, we obtained the cluster information through STRUCTRUE software. Based on the most possible true value of K (K = 3), we described the most prevalent haplogroup C3.3 in Chinese strains (82.1%, 23/28, green color in Figure 1, k = 3). The predominant haplogroup was equal to Chinese 1 (ToxoDB#9) strains which were identified in RFLP technique. The other two haplogroups, C3.1 shown as red, corresponds to archetypical Type I (ToxoDB#10), and C3.2 shown as blue, carries Type II/III and atypical (ToxoDB#205) isolates. Wh6 strain was clusterd in haplogroup C3.3, and was indistinguishable from other virulent strains through MS typing.

When compared to other continental isolates, in spite of existing identical haplogroup with others (Figure 2, k = 4, designated as blue and green), Chinese isolates exhibited a very unique and dominant lineage (presented as red) in clustering results. This grouping data explicitly declared that the major haplotype of *T. gondii* in China was different from other continental strains. South American isolates, previously described as having higher polymorphism, did not show the diversity here, the possible reason for that is the limited number of strains incorporated in this data.

The transition from foraging to agriculture thousands of years ago accelerated the expansion of *T. gondii* in the world [35,36]. Taking the effect of human activity into account, it seems that China might be one of the primary origin spots for *T. gondii* circulation because the ancient country is well known for its oldest and unique civilization with primitive agriculture and animal husbandry, thus anthropization could pressure genetic diversity in *T. gondii* populations [34]. Obviously, more wild type isolates and further studies need to be carried out on such hypothesis in China.

In Figure 4, we obtained phylogenetic trees of Chinese isolates and aligned typical reference strains from a Neighbor-Net phylogenetic network. Instead of constructing a strictly bifurcating topology in a conventional single phylogenetic tree, Splits Tree makes a phylogenetic network with reticulations. A phylogenetic network is superior to the routinely bifurcating phylogeny in describing and visually presenting complicated relationships in population biology. In the present study, the evolutionary tree revealed the microsatellite marker of *W35* showed more divergent features than others. The reticulate results, no matter what type of markers were used, uniformly displayed a highly clonal lineage in Chinese isolates, although a certain degree of recombination was also found here.

Virulence of *T. gondii* is generally defined in the mouse model after intraperitoneal injection of given numbers of tachyzoites. However, due to the diversity of *T. gondii* genomic background, it is difficult to definitely draw global conclusions about virulence [37]. Our prior studies showed that cystogenic and less virulent strains exist in Chinese 1 type [12]. Considering 10 markers in RFLP once used, we evaluated 15 high resolution MS markers for better understanding of the potentially divergent genetic background. Unfortunately, no obvious genotypic difference has been found in the same haplotype Wh6 and Wh3 isolates (Additional file 3). To illuminate virulence differences, we performed the mouse virulence tests with a series of inoculum doses to observe the mortality of infected mice. As a result, mice inoculated with Wh6 had a longer survival than those with the other Chinese isolates (Table 2, Figure 5).

In Figure 6, we mainly focused on Wh3 and Wh6 to evaluate the cell penetration and duplication properties *in vitro.* At defined points post infection, the two isolates showed a similar cell invasion. However, the numbers of tachyzoite multiplication in each infected cell differ in the two isolates, indicating that Wh6 had a lower duplication rate (Figure 6). Previous reports also showed that isolates with low replication and less pathogenicity for mice usually generate more tissue cysts than those with the rapidly dividing strains [38]. The low growth rate and lower virulence of Wh6 may account for the predisposition to form tissue cysts resulting in host latent infection.

The virulence-associated factors are those molecules that are secreted/excreted by the parasites responsible for their invasion, replication, egression, and immunomodulation. We compared the expression of VFs to explore the reason for different phenotypes with the common genotypes of Wh3 and Wh6 strains. Interestingly, we found that the transcriptional level of GRA3 of Wh6 was dramatically higher in the tachyzoites, and dormant bradyzoites as well, than of Wh3 (Figure 7). GRA3 is known to play an important role in shaping parasitophorous vacuole membranes [39,40]. Furthermore, GRA3 has been reported to interact with host cell calcium-modulator and cyclophilin ligand (CAML) of endoplasmic reticulum integral membrane protein [41], which could induce anti-apoptosis [42,43], suggesting a potential role of GRA3 in regulation of apoptosis-associated cell process.

## Conclusions

Microsatellite genotyping and phylogenetic networking displayed a very limited diversity of *T. gondii* isolates in China. The results indicate that the MS genotyping is a useful tool with high resolution and simplicity in discovering the genetic divergency of *Toxoplasma gondii*, although it is insufficient to distinguish less virulent strains from high virulent ones in Chinese 1 genotype.

## Competing interests

The authors declare that they have no competing interests.

## Authors’ contributions

JLS, ML and ZRL conceived and designed the study. ML, XWM, HC, LW, HQW and AMZ performed the experiments. QLL, WW, FLL and JD analyzed the data. ML and JLS drafted the manuscript. All authors read and approved the final manuscript.

## Supplementary Material

Additional file 1 **PCR and sequencing primers using for phylogenetic analysis of *T. gondii*.** Click here for file

Additional file 2 **Primers for qRT-PCR of virulence-associated factors of *T. gondii.***
Click here for file

Additional file 3 Genotyping results of 28 Chinese isolates and 6 reference strains with 15 microsatellite markers in a single multiplex PCR assay. Click here for file
