# Supplementary material for: Phylogeny and virulence divergency analyses of Toxoplasma gondii isolates from China
Source: Parasit Vectors. 2014 Mar 28;7:133. doi: 10.1186/1756-3305-7-133 (PMC3986613; doi:10.1186/1756-3305-7-133)
Supplement: Additional file 1 — PCR and sequencing primers using for phylogenetic analysis of T. gondii . [file 1756-3305-7-133-S1.doc]

Additional file 1. PCR and sequencing primers using for phylogenetic analysis of *T. gondii*

| **Locus** | **Intron** | **Chr** | **bp** | **PCR-primers (5’-3’)** | **Sequencing primers (5’-3’)** |
| --- | --- | --- | --- | --- | --- |
| *W35* |  | Ⅱ | 242 | F：GGTTCACTGGATCTTCTCCAA  R：AATGAACGTCGCTTGTTTCC | F：GGTTCACTGGATCTTCTCCAA  R：AATGAACGTCGCTTGTTTCC |
| *UPRT* | Intron1 | Ⅺ | 467 | F：CCCGATATTCGACAAACGAC | F：CCCGATATTCGACAAACGAC |
|  |  |  |  | R：GAGCCGTCTGCTTCATGAGC | R：GAGCCGTCTGCTTCATGAGC |
|  | Intron7 | Ⅺ | 459 | F：TCTTGTTTGCTTTCCTCGGC | F：TGGTCGTCGTCACTTGTTA |
|  |  |  |  | R：CGGTCAGCGGTCTGTCAAAA | R：GCAGCCTCACAACTAAAACT |
| *MIC 2* | Intron1 | Ⅶa | 413 | F：TTCGGTCTGATGTTCGCTTG | F：CTTGCGGTATGTGGATGTGG |
|  |  |  |  | R：GGGCAACACCATTAAAAAGG | R：GTATTCCTATGCTTCCAGAC |
|  | Intron3 | Ⅶa | 371 | F：GGTTCTGATGAAAACGAGAC | F：GAGAACAGTCCAAGAAAGAG |
|  |  |  |  | R：CTACTCCATCCACATATCAC | R：ATCACTATCGTCATCCACGG |
| *BTUB* | Intron1 | Ⅸ | 465 | F：AGGTCATCTCGGACGAACAC | F：GTTTTCCCTGTGGAGAATC |
|  |  |  |  | R：TTCTCTCCAGCTGCAAGTCA | R：GCGAACAGGAGTGAAGTATT |
|  | Intron2 | Ⅸ | 141 | F：GCCATCCAGGAGATGTTCAA | F：GTGTGTCCGACCAATTCACA |
|  |  |  |  | R：CTGCTGATACTCGGACACCA | R：CTCGGTGAATTCCATCTCGT |
| *EF1* | Intron1 | Ⅹ | 647 | F：AAATGCACCCTTTTCTTAAA | F：AAATTGTCCCGCCATCAG |
|  |  |  |  | R：CACATGAAGGTACACCAAAA | R：GATCAACATTCAAGAGGTGGA |
| *HP* | Intron2 | Ⅳ | 817 | F：GACAGAAACACGCAGAGAAT | F：ATAATACAGTCAGTTCCCTCGAT |
|  |  |  |  | R：TAATCTTTGTTCCCATGCTT | R：TTTCTCACCTCAGTCTCTTG |
| *AP1* | Apicoplast genome | | 671 | F：AAAATAACGCGAAAAGATTCA | F：CGTGCAAAACAATCATCAGA |
|  |  |  |  | R：TGTGGAAGATATGGAAATAAAGGA | R：GCAGAAAACGCTGATTTACCTT |
| *AP2* | Apicoplast genome | | 640 | F：TCCTTTATTTCCATATCTTCCACA | F：TTCCACATAATTTATCTCCAACTG |
|  |  |  |  | R：AAAACCTTTAGTATGAAACGGTGAA | R：TGGATATGATTTTGAAGATGCTG |
| *AP3* | Apicoplast genome | | 502 | F：TTCCTCCTGTATGAAAAGTTCG | F：TTTTGAACAAACTGTTCTTCCAC |
|  |  |  |  | R：TGTTATGGAGCAAGAAAAGGAA | R：CAAATGCTGGGTATTTAACACG |
