# Supplementary material for: Phylogeny and virulence divergency analyses of Toxoplasma gondii isolates from China
Source: Parasit Vectors. 2014 Mar 28;7:133. doi: 10.1186/1756-3305-7-133 (PMC3986613; doi:10.1186/1756-3305-7-133)
Supplement: Additional file 2 — Primers for qRT-PCR of virulence-associated factors of T. gondii. [file 1756-3305-7-133-S2.doc]

Additional file 2. Primers for qRT-PCR of virulence-associated factors of *T. gondii*

| **Batch No.** | **VFs** | **Primer sequences (5’-3’)** | **Size (bp)** | **Position** |
| --- | --- | --- | --- | --- |
| 1 | ROP2 | F: CTCAACACACAAGTCCCTCGT | 244 | 213-456 |
|  |  | R: CATTCGTTCCGCTGTAGTCTC |  |  |
| 2 | ROP4 | F: CAAAAGTGGTGGCTGAAAATG | 214 | 2524-2737 |
|  |  | R: CGGCACAATGAGAACAAGAAT |  |  |
| 3 | ROP5 | F: CAGGAACTGGAGACGATTCAG | 106 | 158-263 |
|  |  | R: GATGTGGTGGTAGACGATGCT |  |  |
| 4 | ROP16 | F: CCGACTTCCTTACAGCCTTTC | 105 | 1848-1952 |
|  |  | R: TCGTTACAATCCTTGGCAGAG |  |  |
| 5 | ROP18 | F: ACCGATGAGACTATGCGTGAC | 143 | 980-1122 |
|  |  | R: GATGCTGGCTGTCCCTCTAAC |  |  |
| 6 | GRA2 | F: CATTGTTTGCTGGTTGTTGC | 166 | 16-181 |
|  |  | R: CTGGGAGTGGTGGTGTATGTT |  |  |
| 7 | GRA3 | F: TTCTCGCCGCCTACTACATT | 182 | 634-815 |
|  |  | R: TGTGTCCAATCTGCGTCAAC |  |  |
| 8 | GRA5 | F: GGCGTGAACAACAACAGGTA | 238 | 678-915 |
|  |  | R: CCTTACTCTTCCTCGGCAACT |  |  |
| 9 | GRA7 | F: ACATCTACGAGGAGCACACTGA | 139 | 344-482 |
|  |  | R: ACAAGTCTATCAGCGGCAAAAT |  |  |
| 10 | GRA15 | F: GCTTGGGTATCTTACGGTGCT | 242 | 641-882 |
|  |  | R: CGTTTTCGGTCTGTTCACTCT |  |  |
| 11 | MIC6 | F: GGAAGATGAAGGTGAAAGTCGT | 88 | 804-891 |
|  |  | R: GCTTTCCTCAAGTGGTGTTCTT |  |  |
| 12 | GAPDH | F: GTCTCCTCTGACTTCGTCCACT | 118 | 892-1009 |
|  |  | R: AGTATCCCCACTCGTTGTCGTA |  |  |
