# Supplementary material for: Phylogeny and virulence divergency analyses of Toxoplasma gondii isolates from China
Source: Parasit Vectors. 2014 Mar 28;7:133. doi: 10.1186/1756-3305-7-133 (PMC3986613; doi:10.1186/1756-3305-7-133)
Supplement: Additional file 3 — Genotyping results of 28 Chinese isolates and 6 reference strains with 15 microsatellite markers in a single multiplex PCR assay. [file 1756-3305-7-133-S3.doc]

Additional file 3. Genotyping results of 28 Chinese isolates and 6 reference strains with 15 microsatellite markers in a single multiplex PCR assay

| **Strain** | **Host** | **Haplotype** | **Genotype** | ***TUB2*** | ***W35*** *^a^* | ***TgM-A*** | ***B18*** | ***B17*** | ***M33*** | ***Ⅳ.1*** | ***Ⅺ.1*** | ***M48*** | ***M102*** | ***N60*** | ***N82*** | ***AA*** | ***N61*** | ***N83*** |
| --- | --- | --- | --- | --- | --- | --- | --- | --- | --- | --- | --- | --- | --- | --- | --- | --- | --- | --- |
| ENT | France(human) | C3.1 | #10 ( TypeⅠ) | 291 | 248 | 209 | 160 | 342 | 169 | 274 | 358 | 209 | 166 | 145 | 119 | 267 | 87 | 306 |
| RH | USA (human) | C3.1 | #10 ( TypeⅠ) | 291 | 248 | 209 | 160 | 342 | 169 | 274 | 358 | 209 | 166 | 145 | 119 | 265 | 87 | 306 |
| ME49 | USA(sheep) | C3.2 | #1( Type Ⅱ) | 289 | 242-2 | 207 | 158 | 336 | 169 | 274 | 356 | 215 | 174 | 142 | 111 | 265 | 91 | 310 |
| PTG | USA(sheep) | C3.2 | #1( Type Ⅱ) | 289 | 242-2 | 207 | 158 | 336 | 169 | 274 | 356 | 215 | 174 | 142 | 111 | 265 | 91 | 310 |
| PRU | France (human) | C3.2 | #1( Type Ⅱ) | 289 | 242-2 | 207 | 158 | 336 | 169 | 274 | 356 | 209 | 176 | 142 | 117 | 265 | >121 | 310 |
| VEG | USA(human) | C3.2 | #2(Type Ⅲ ) | 289 | 242-3 | 205 | 160 | 336 | 165 | 278 | 356 | 213 | 188 | 153 | 111 | 267 | 89 | 312 |
| TgCtwh1 | China（cat） | C3.3 | #9 (Chinese 1) | 293 | 242-1 | 211 | 160 | 336 | 169 | 274 | 354 | 215 | 172 | 145 | 127 | 289 | 91 | 308 |
| TgCtwh2 | China（cat） | C3.3 | #9 (Chinese 1) | 293 | 242-1 | 211 | 160 | 336 | 169 | 274 | 354 | 215 | 172 | 145 | 127 | 289 | 91 | 308 |
| TgCtwh3 | China（cat） | C3.3 | #9 (Chinese 1) | 293 | 242-1 | 211 | 160 | 336 | 169 | 274 | 354 | 215 | 172 | 145 | 111 | 273 | 93 | 306 |
| TgCtwh4 | China（cat） | C3.3 | #9 (Chinese 1) | 293 | 242-1 | 211 | 160 | 336 | 169 | 274 | 354 | 217 | 172 | 145 | 127 | 295 | 91 | 308 |
| TgCtwh5 | China（cat） | C3.3 | #9 (Chinese 1) | 293 | 242-1 | 213 | 160 | 336 | 169 | 274 | 354 | 217 | 172 | 143 | 127 | 287 | 91 | 308 |
| TgCtwh6 | China（cat） | C3.3 | #9 (Chinese 1) | 293 | 242-1 | 211 | 160 | 336 | 169 | 274 | 354 | 215 | 172 | 145 | 111 | 287 | 93 | 306 |
| TgCtwh8 | China（cat） | C3.3 | #9 (Chinese 1) | 293 | 242-1 | 211 | 160 | 336 | 169 | 274 | 354 | 215 | 172 | 145 | 121 | 283 | 95 | 308 |
| TgCtwh10 | China（cat） | C3.3 | #9 (Chinese 1) | 293 | 242-1 | 213 | 160 | 336 | 169 | 274 | 354 | 215 | 172 | 143 | 113 | 291 | 99 | 308 |
| TgCtwh11 | China（cat） | C3.3 | #9 (Chinese 1) | 293 | 242-1 | 211 | 160 | 336 | 169 | 274 | 354 | 215 | 172 | 145 | 121 | 297 | 91 | 308 |
| TgCtwh12 | China（cat） | C3.3 | #9 (Chinese 1) | 293 | 242-1 | 211 | 160 | 336 | 169 | 274 | 354 | 215 | 172 | 145 | 121 | 297 | 91 | 308 |
| TgCtwh14 | China（cat） | C3.3 | #9 (Chinese 1) | 293 | 242-1 | 211 | 160 | 336 | 169 | 274 | 354 | 215 | 172 | 145 | 125 | 297 | 91 | 308 |
| TgCtwh19 | China（cat） | C3.3 | #9 (Chinese 1) | 293 | 242-1 | 211 | 160 | 336 | 169 | 274 | 354 | 215 | 172 | 145 | 121 | 297 | 91 | 308 |
| TgCtxz2 | China（cat） | C3.3 | #9 (Chinese 1) | 293 | 242-1 | 211 | 160 | 336 | 169 | 274 | 354 | 215 | 172 | 145 | 113 | 285 | 91 | 308 |
| TgCtxz4 | China（cat） | C3.3 | #9 (Chinese 1) | 293 | 242-1 | 211 | 160 | 336 | 169 | 274 | 334 | 215 | 172 | 145 | 111 | 291 | 95 | 306 |
| TgCtxz6 | China（cat） | C3.3 | #9 (Chinese 1) | 293 | 242-1 | 211 | 160 | 336 | 169 | 274 | 354 | 215 | 172 | 145 | 111 | 297 | 95 | 306 |
| TgCtgd1 | China（cat） | C3.3 | #9 (Chinese 1) | 293 | 242-1 | 211 | 160 | 336 | 169 | 274 | 354 | 211 | 172 | 145 | 125 | 297 | 91 | 308 |
| TgCtgd2 | China（cat） | C3.3 | #9 (Chinese 1) | 293 | 242-1 | 211 | 160 | 336 | 169 | 274 | 354 | 215 | 172 | 145 | 123 | 291 | 91 | 308 |
| TgCtsx1 | China（cat） | C3.3 | #9 (Chinese 1) | 293 | 242-1 | 211 | 160 | 336 | 169 | 274 | 354 | 219 | 170 | 130 | 115 | 279 | 99 | 308 |
| TgCtsx2 | China（cat） | C3.3 | #9 (Chinese 1) | 293 | 242-1 | 211 | 160 | 336 | 169 | 274 | 354 | 219 | 170 | 130 | 115 | 279 | 99 | 308 |
| TgCtys1 | China（cat） | C3.3 | #9 (Chinese 1) | 293 | 242-1 | 211 | 160 | 336 | 169 | 274 | 354 | 221 | 172 | 145 | 111 | 285 | 97 | 306 |
| TgCtys2 | China（cat） | C3.3 | #9 (Chinese 1) | 293 | 242-1 | 211 | 160 | 336 | 169 | 274 | 354 | 215 | 172 | 145 | 127 | 291 | 91 | 308 |
| TgCtgy1 | China（cat） | C3.3 | #9 (Chinese 1) | 293 | 242-1 | 213 | 160 | 336 | 169 | 274 | 354 | 215 | 172 | 130 | 111 | 273 | 93 | 306 |
| gpkfx171 | China（pork） | C3.3 | #9 (Chinese 1) | 293 | 242-1 | 211 | 160 | 336 | 169 | 274 | 354 | 215 | 172 | 145 | 125 | 285 | 91 | 308 |
| TgCtxz3 | China（cat） | C3.2 | #205(Atypical) | 289 | 242-2 | 207 | 160 | 346 | 169 | 274 | 356 | 213 | 174 | 140 | 129 | 259 | 107 | 306 |
| TgCtxz5 | China（cat） | C3.2 | #205(Atypical) | 289 | 242-2 | 207 | 160 | 346 | 169 | 274 | 356 | 213 | 174 | 140 | 129 | 259 | 107 | 306 |
| TgCtxz7 | China（cat） | C3.2 | #205(Atypical) | 289 | 242-2 | 207 | 160 | 346 | 169 | 274 | 356 | 213 | 174 | 140 | 129 | 259 | 107 | 306 |
| TgCtxz8 | China（cat） | C3.2 | #205(Atypical) | 289 | 242-2 | 207 | 160 | 346 | 169 | 274 | 356 | 213 | 174 | 140 | 129 | 259 | 107 | 306 |
| TgCtxz1 | China（cat） | C3.1 | #10 (TypeⅠ) | 291 | 248 | 209 | 160 | 342 | 169 | 274 | 358 | 209 | 166 | 145 | 119 | 265 | 89 | 306 |

*^a^* 242-1 for allele of (TC)_4_ CC(TC)_2_ (TG)_2_ , 242-2 for allele of (TC)_7_ (TG)_2_ , 242-3 for allele of (TC)_6_ (TG)_3_
